# Numerical Investigation of Dimensionless Parameters in Carangiform Fish Swimming Hydrodynamics

**DOI:** 10.3390/biomimetics9010045

**Published:** 2024-01-11

**Authors:** Marianela Machuca Macías, José Hermenegildo García-Ortiz, Taygoara Felamingo Oliveira, Antonio Cesar Pinho Brasil Junior

**Affiliations:** 1 Department of Mechanical Engineering and Industrial Design, Faculty of Engineering, University of Cadiz, Puerto Real, 11519 Cadiz, Spain; mere.garcia@uca.es; 2 Laboratory of Energy and Environment, Department of Mechanical Engineering, University of Brasilia, Brasília 70910, DF, Brazil; taygoara@unb.br (T.F.O.); brasiljr@unb.br (A.C.P.B.J.)

**Keywords:** swimming fish, quasi-propulsive efficiency, Reynolds number, Strouhal number, slip number, leading-edge vortex, wake vortex, CFD, biomimetics

## Abstract

Research into how fish and other aquatic organisms propel themselves offers valuable natural references for enhancing technology related to underwater devices like vehicles, propellers, and biomimetic robotics. Additionally, such research provides insights into fish evolution and ecological dynamics. This work carried out a numerical investigation of the most relevant dimensionless parameters in a fish swimming environment (Reynolds Re , Strouhal St , and Slip numbers) to provide valuable knowledge in terms of biomechanics behavior. Thus, a three-dimensional numerical study of the fish-like lambari, a BCF swimmer with carangiform kinematics, was conducted using the URANS approach with the *k*- ω -SST transition turbulence closure model in the OpenFOAM software. In this study, we initially reported the equilibrium Strouhal number, which is represented by $St^{*}$ , and its dependence on the Reynolds number, denoted as Re . This was performed following a power–law relationship of $St\propto Re^{\left(-\alpha \right)}$ . We also conducted a comprehensive analysis of the hydrodynamic forces and the effect of body undulation in fish on the production of swimming drag and thrust. Additionally, we computed propulsive and quasi-propulsive efficiencies, as well as examined the influence of the Reynolds number and Slip number on fish performance. Finally, we performed a vortex dynamics analysis, in which different wake configurations were revealed under variations of the dimensionless parameters St , Re , and Slip . Furthermore, we explored the relationship between the generation of a leading-edge vortex via the caudal fin and the peak thrust production within the motion cycle.

## 1. Introduction

Bio-inspired devices have gained significant attention recently due to their potential for solving complex engineering problems [1,2,3]. Among them, we can find flying devices, swimmers, etc. Many researchers have focused on studying fish swimming to understand the underlying principles and mechanics that can be applied to the design and development of biomimetics devices like autonomous underwater vehicles (AUVs) [4,5]. AUVs offer numerous benefits when used for challenging tasks. One of the key advantages is their ability to operate independently, thus eliminating the need for human operators to be physically present underwater or on a nearby vessel [6,7].

Thus far, despite technological advances and artificial intelligence, modern robots have not yet been able to replicate some of the specific characteristics of fish movement, such as maneuverability or flexibility. Moreover, from a propulsion point of view, there is still a long way to go in terms of the correct estimation of efficiency, which will have to be achieved by taking into account the difficulty of decoupling thrust from drag [8]. For a highly adaptive and cost-effective technology, it is crucial to investigate the characteristics of fish that have developed their shapes, swimming techniques, and sensory capabilities over many years of evolution [9,10,11]. By studying the parameters involved in fish swimming, such as dimensionless numbers like Reynolds [12], Strouhal [13,14], Slip [15], or swimming [10]—along with hydrodynamic efficiency, drag, and power coefficients [8,14,16,17]—engineers and biologists can obtain valuable information for the design and performance of bio-inspired swimming devices.

The study of body caudal fin (BCF) swimming motion has been a recurring topic in the literature, as 85% of fish species employ this swimming mode, as noted by Wei [18]. This mode relies on body and caudal fin undulation to generate propulsive thrust force. BCF swimmers are categorized into several modes, including anguilliform, sub-carangiform, carangiform, and thunniform. The primary distinctions among BCF swimming mechanisms are rooted in kinematic aspects, such as the amplitude wave envelope and wavelength, as well as the forces involved in thrust generation [9]. Thunniform swimming involves vorticity flow around the fish, and it utilizes a lift-based method. On the other hand, anguilliform, sub-carangiform, and carangiform modes are associated with reaction forces that generate acceleration, which is achieved by considering the added mass effect [19]. Distinctively, the vorticity effect on force production is also observed in carangiform fish [20,21].

Regarding body undulation, anguilliform swimmers tend to exhibit longer undulation wavelengths during locomotion. In contrast, carangiform swimmers typically have shorter undulation wavelengths, in which they undulate approximately one-third of their body length when swimming [9]. The BCF swimming modes are highly efficient and versatile, enabling fish to achieve high speeds and maneuverability in various aquatic environments [22,23]. Therefore, gaining a comprehensive understanding of the kinematics, efficiency, and wake behavior induced by these swimmers contributes to developing bio-inspired devices [4,19,24].

Previous studies in the field of fish swimming biomechanics have utilized experimental and numerical approaches to analyze locomotion forces, power consumption, hydrodynamic efficiency, and wake vortices. Experimental studies often employ methods such as particle image velocimetry using live or robotic fish [25,26,27,28,29,30]. Numerical simulations have been conducted employing various techniques, including panel methods with potential flow assumptions for laminar or inertial regimes [25,31,32], the immersed boundary method [15,33,34,35,36,37], as well as the combined level set/immersed boundary method developed by Cui et al. [22] and Tekkethil et al. [26]. Other studies have employed unsteady Reynolds-average Navier–Stokes equations to address three-dimensional viscous flow [38], in which they employed various turbulence models to address turbulence closure problems. For instance, Chang et al. [39], Li et al. [40], and Macias et al. [13] utilized the *k*- ω -SST model to investigate carangiform and thunniform swimmers, while Adkins and Yan [41] employed the *k*- ϵ model to analyze the flow around fish bodies that exhibited carangiform kinematics within a viscous flow. In contrast, a few studies have adopted the large eddy simulation (LES) turbulent approach, which resolves large turbulence scales while modeling only the smaller ones. For example, Bottom et al. [42] examined the hydrodynamics of stingrays, and Ogunka et al. [43] investigated the ground effects on the swimming behavior of eel-like fish.

In the context of hydrodynamic efficiency and propulsive force calculation, the literature emphasizes the difficulty of determining the net efficiency of a self-propelled body swimming at a constant velocity where the hydrodynamic net force must be zero (thus balancing drag and thrust). This challenge becomes even more complex when trying to compute propulsive efficiency using the thrust generated by an undulating body, as it introduces the complication of distinguishing between thrust and drag forces within such a dynamic system [8,15].

Some authors have employed inviscid methodologies to estimate the thrust and the power generated by swimming fish in high Reynolds numbers, such as the elongated body theory (EBT) proposed by Lighthill’s model [12,25,44,45,46]. However, some works have argued that the thrust is overestimated [15,47]. Other authors, like Borazjani and Sotiropoulos [15], have employed computational fluid dynamics (CFDs) techniques to decouple drag and thrust forces, which is achieved by considering thrust as the mean of the positive part of the undulating wave, given its periodic nature with a zero mean. However, Maertens et al. [8] reported that the method has the limitation that two gaits with the same swimming power and speed will artificially have different efficiencies if their drag trace is different.

Numerous investigations have been conducted on the debate surrounding drag increase or decrease due to body undulations without the reaching of a consensus, thus making it an ongoing topic in the literature. Authors such as Barrett et al. [31] reported a reduction in drag with undulations at high Reynolds numbers, which is reminiscent of Gray’s paradox [48]. On the other hand, other researchers have endorsed drag enhancement caused by undulations, both in potential flows [47,49] and in flows with Reynolds numbers at the order of $10^{3}$ – $10^{4}$ [8,50]. In this way, the Bone–Lighthill boundary layer thinning hypothesis [49] suggests that the thinning of a boundary layer due to undulatory motion results in increased viscous drag in swimming fish, thereby generating a swimming drag that is 3 to 5 times greater than rigid body drag. In this way, Anderson et al. [51] also did not find conclusive results, reporting that they could not ensure the same flow conditions in both cases (i.e., in deformed and non-deformed fish). In our work, the Borazjani and Sotiropoulos method [15] was employed to separate the drag and thrust forces, thereby ensuring accurate calculations. It was observed that the calculated drag force was solely attributable to friction forces that were computed directly from simulations. This approach contributes to the discussion on drag production in undulatory motions, thus eliminating any inconvenience derived from the method.

In terms of efficiency calculation, to address the challenge of separately calculating thrust and drag, Maertens et al. [8] defined quasi-propulsive efficiency as the ratio of the power required to tow a body in a rigid-straight condition to the power needed for self-propulsion, where both measured at the same speed. The authors reported that quasi-propulsive efficiency is the most suitable dimensionless quantity for defining the best propulsion system for a given body and velocity. Other investigators have employed the definition of propulsive efficiency as a rational fitness indicator. For example, Maertens et al. [52] conducted 3D simulations for a danio-like fish at a Reynolds number of 5000, and Li et al. [53] analyzed the impact of median fins on hydrodynamics in carangiform swimming. Additionally, Cui et al. [22] reported an increase in swimming efficiency concerning tail–beat frequency and an amplitude coefficient at Re = 185.

The influence of the Reynolds number on hydrodynamic efficiency metrics is not very clear in the literature because the employed definition of efficiency appears to be decisive. On the one hand, Wei et al. [18] emphasized that a necessary condition for achieving highly efficient propulsion is to maintain a swimming state with a low Reynolds number and a high Strouhal number. On the other hand, Maertens et al. [8] reported a weak dependence on the Reynolds number, with a 7% difference in the efficiency calculation for Reynolds numbers of 5000 and 2500. Additionally, there are studies in which fish movement was induced by pitching an airfoil or a flat plate, where the researchers established an increase in efficiency by increasing the Reynolds number [54,55,56]. This was attributed to the fact that fish often reach high swimming speeds, which are characterized by high Reynolds numbers ( Re > $10^{4}$ ). This was ascertained by placing them in an inertial regime, where viscous forces have a reduced impact and where inertial forces dominate the dynamics of movement [15,57,58]. In this study, hydrodynamic efficiency measurements were conducted in an extensive range of Reynolds numbers ( $10^{3}$ – $10^{4}$ ) to carangiform swimmers through three-dimensional viscous numerical simulations in order to contribute to the discussion in the literature.

Besides the Re , another parameter identified in the literature with a significant impact on the efficiency is the slip number, which represents the ratio of swimming velocity to the phase velocity of body wave undulation. As the Slip number decreases, the power consumption increases, thus indicating a decrease in propulsive efficiency [16,59]. This effect is because the Slip number strengthens the effect of the low-pressure kernel, thus leading to an increase in power consumption [59]. On the other hand, in the eyes of the slender body theory, an excessive increase in the Slip number would cause a worsening of maneuverability in executing longitudinal movements and lateral maneuvers [16].

Furthermore, it is crucial to emphasize that propulsive performance is influenced not only by frictional effects, but also by the generation and interaction of vortex dynamics. Therefore, the study of vortex dynamics is also a pertinent aspect for analyzing the hydrodynamic performance of a fish.

As previously mentioned, carangiform swimming with undulating wave-like movements generates vortices due to the body and caudal fin striding from side to side. These vortices can contribute to thrust production, thus influencing the efficiency of the aquatic propulsion [13]. In the past, research regarding the vortices generated by fish swimming has primarily centered on examining trailing wakes, where both traditional and reverse von Kármán vortex streets have been identified. The reverse von Kármán vortex street is representative of the conventional wake pattern observed during fish swimming in their natural environment, which results in the generation of thrust through the expulsion of high-velocity fluid. During this process, the fish ejects positive vortices in a counterclockwise direction above the fish’s midplane, while negative vortices, through rotating clockwise, are found below it [13,15].

More recently, researchers have been studying the exploration of the reattachment mechanism of leading-edge vortices (LEV), a phenomenon that has been well documented in insect and bird flight for its role in enhancing propulsive forces [60], though also particularly within the domain of fish biomechanics. Borazjani and Daghooghi [61] were among the pioneering investigators who conducted a comprehensive examination of the LEV associated with the caudal fins of fish. Their investigation focused on elucidating the mechanisms through which these vortices generated locomotive forces under diverse swimming frequencies and across various flow regimes, including transitional and inertial. Their research discerned the distinctions between stable and detached LEVs within both inertial and transitional flow regimes, and they offered valuable insights into the influence of fish swimming frequencies. Subsequently, Liu et al. [34] verified the presence of LEVs in the context of carangiform swimmers. Their work analyzed, in detail, the intricate interactions between the LEV and other vortices, such as the trailing edge vortex (TEV) and the posterior body vortex (PBV). Moreover, Bottom et al. [42] made a noteworthy contribution by identifying the existence of a leading-edge vortex on the pectoral disk of fast-swimming stingrays. This vortex induced a low-pressure region within the fast-swimming stingray’s hydrodynamic environment.

Brooks and Green [29] conducted experimental research into the vortex dynamics generated by a two-degree-of-freedom fish model. On the other hand, Mignano et al. [62] employed multiple fins to engineer propulsive forces for swimming, and they discerned the characteristics of the attached LEV and its contribution to thrust generation. Conversely, Macias et al. [13] presented a numerical investigation into the temporal evolution of the LEV induced by a tuna exhibiting fish-like characteristics. Several other studies have focused on examining the influence of tail geometry on the properties of a leading-edge vortex. Xiong and Liu [63] analyzed three distinct forked caudal fins with varying chord lengths, and they reported a direct relationship between the LEV and the angle of attack (AoA). Han et al. [64] reported a systematic study of the effects of dorsal/anal fin shapes and the flapping phase on the hydrodynamic performance of a bluegill sunfish model, whereby they numerically evaluated the median-fin interactions (MFI) and leading-edge vortex production. Lastly, Tack and Gemmell [65] carried out a comprehensive comparative experimental study by comparing forked and truncated tail shapes. Their work encompassed examining the fluid mechanical properties, including those related to the LEV.

In engaging with the scientific literature, the pursuit of assessing the efficiency of fish swimming and its correlation with vortex production is achieved through systematic variations of the pivotal dimensionless parameters governing this phenomenon, and this remains indispensable for advancing our comprehension of fish hydrodynamics. It also underscores the untapped potential in extracting insights from the inherent swimming mechanisms of fish. Such insights, in turn, can contribute to the ecological understanding of fish swimming and the enhancement of artificial propulsion systems, such as UAVs, as well as the design of bio-inspired devices, including robots.

In our research, we investigated the influence of Re , St , and Slip numbers on force productions and hydrodynamic efficiencies. This goal was further motivated by the ongoing discourse surrounding the quest for an optimal flow regime and kinematic profile in undulation modes, where the ultimate purpose is to achieve optimal hydrodynamic performance. Additionally, our investigation will encompass the intricate dynamics of vortex generation in fish swimming, thus broadening the horizons of our understanding in this domain. Our findings are presented to contribute to the discussion about drag production in carangiform undulatory motion, where the decoupling of drag and thrust forces are emphasized. Additionally, the evaluation of different types of efficiencies and wake vortex dynamics is introduced for a wide range of Reynolds numbers and slip values, and this is conducted through three-dimensional viscous flow numerical simulations.

The present paper is structured as follows: Section 2 provides a comprehensive description of fish swimming kinematics, elucidates the key dimensionless parameters pertinent to the study, and outlines various efficiency metrics. It also details the numerical methodology employed for conducting fish swimming simulations. Section 3 presents and discusses the results of the hydrodynamic efficiency and vortex dynamics. Finally, in Section 4, we present the main conclusions of the study.

## 2. Methodology

### 2.1. Fish Swimming Characteristics

The species considered for investigation is the lambari (*Astyanax bimaculatus*), a common fish found in Brazilian rivers. The fish body and caudal fin surfaces are generated using elliptical and hydrofoil cross-sections, respectively. The lambari morphometric data were extracted from Botelho et al. [66], where the fish’s total length was L=0.12 m, the distance from the head to the caudal peduncle was $L^{\prime }=0.09$ m, and the aspect ratio was AR=1.1 (the ellipse aspect ratio is the relation between the mayor and minor axis, where the mayor axis is the fish height) [13].

**Figure 1:**
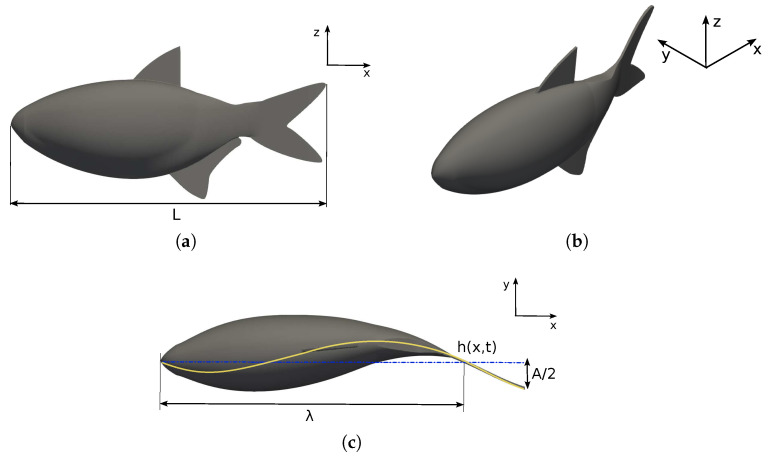
Lamabri geometry: (**a**) frontal, (**b**) perspective, and (**c**) top views.

**Figure 2:**
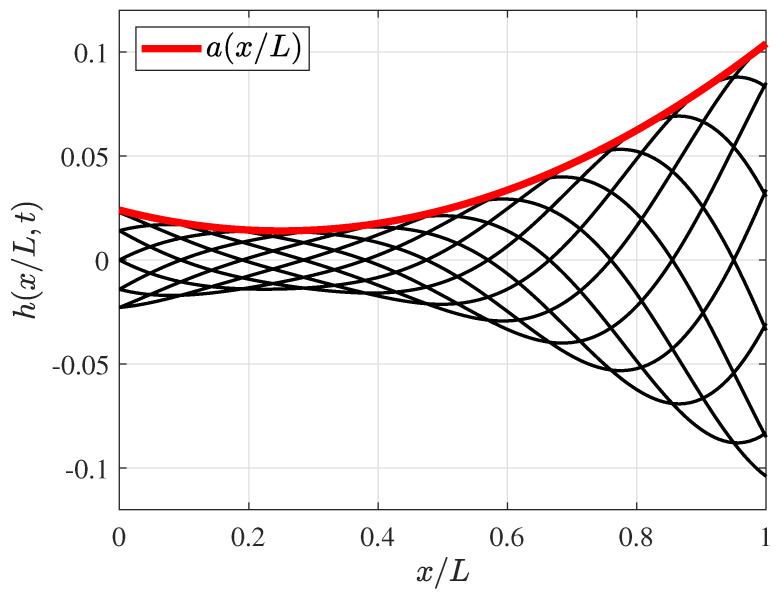
Non−dimensional fish midline deformation h(x/L,t) in a period (*T*), where a time step ( T/10 ) and wave amplitude function envelope a(x/L) is considered in the red line.

Concerning the kinematics, as reported by Macias et al. [13], the lambari is a carangiform swimmer. The fish movement is based on the BCF propulsion mode, where the thrust force is due to the body’s undulation in a propelling wave. Carangiform fish swimming kinematics are described by a sinusoidal equation with variable amplitude such as Equation (1), where h(x,t) is the fish’s midline displacement that reproduces the lateral undulation of the fish body at time *t* (see Figure 1c and Figure 2).

(1) h(x,t)=a(x)sin(kx−ωt).

In Equation (1), k=2π/λ is the tail wave number, where λ is the wavelength of the propulsive traveling wave, ω=2πf is the characteristic swimming frequency ( rad/s ), and a(x) is the variable amplitude of the envelope defined by $a\left(x\right)=a_{0}+a_{1}x+a_{2}x^{2}$ , whereby $a_{0}=0.02$ , $a_{1}=-0.08$ , and $a_{2}=0.16$ , and the experimental coefficients are those as reported by [67]. Subsequently, the authors defined the maximum amplitude value based on the amplitude function such as $a_{max}=0.1$ ; thus, $h_{max}=0.1L$ , and the maximum tail displacement is $A=2h_{max}$ , whereas, in this analysis, A=0.024 . The dimensionless wavelength was initially chosen as λ/L=95% (as proposed by Borazjani and Sotiropoulos [15]), which is in the range of 89–110%, as was observed in most carangiform swimmers by Videler and Wardle [67]. Later, the wavelength was varied, as reported further in this paper. Figure 2 presents the amplitude envelope a(x/L) and fish’s midline deformation h(x/L,t) functions as dimensionless with a fish length body.

In this study, hydrodynamic efficiency and vortex production are investigated under different flow regimes (Reynolds number, Re ), and fish swimming frequencies (Strouhal number, St ). In addition, the slip number influence is analyzed to understand the kinematic relationship between the forward velocity *U* and the phase velocity of the undulation wave that propagates backward along the fish body ( fλ ).

(2) Re=UL/ν;St=fA/U;Slip=U/fλ,

where *L* is fish length, *U* is the undisturbed flow velocity, ν is the water kinematic viscosity, *f* is the characteristic frequency of swimming ( $s^{-1}$ ), and *A* is the amplitude of the tail stride in a half cycle.

### 2.2. Hydrodynamic Efficiency Metrics, Power Consumption, and Hydrodynamic Forces

Fish achieve their propulsive motion by undergoing lateral deformations that displace a certain volume of fluid, which thus facilitates their movement. The power generated during swimming consists of two components: one is known as useful power, which is responsible for the fish’s locomotion; and the other is associated with the lateral deformations the fish makes to move, which are considered as losses and are closely related to the fish’s swimming mode. In this work, the problem is approached from a hydrodynamic perspective, whereby the mechanical efforts in the fish–flow interactions during swimming are solely quantified without considering biological characteristics such as muscular energy or oxygen consumption, as previously mentioned by Maertens et al. [8].

Efficiency is, therefore, defined as the ratio between the useful power, $P_{u}\left(t\right)$ , and the total power that the fish expends for its locomotion, $P_{t}\left(t\right)$ , such as

(3) $$\eta =\frac{P_{u}\left(t\right)}{P_{t}\left(t\right)}.$$

The instantaneous total power $P_{t}\left(t\right)$ is given by the integral over the surface of the fish of the product of hydrodynamic forces, F(t) , and the velocity at the boundary, $v_{i}$ , is defined in Equation (4), such as

(4) $$P_{t}\left(t\right)=\int_{A}F_{i}\left(t\right)\cdot v_{i}\left(t\right)dA.$$

In this study, measurements were conducted over a specific time interval, namely one swimming cycle, where the mean values of power represented by $\bar{P_{u}}$ and $\bar{P_{t}}$ are computed from the time-averaged instantaneous power.

First, one might consider defining swimming efficiency as a net efficiency where $\bar{P_{u}}$ is calculated as the product of the net thrust force in the direction of motion and the fish’s translational velocity $\bar{P_{u}}=\bar{F_{T_{N}}}U$ . However, in the situation where the fish is swimming at a constant speed, this force is null ( $\bar{F_{T_{N}}}=0$ ) since the thrust and drag forces are balanced. This is the scenario analyzed in this study, where the fish swim steadily. Therefore, this definition of efficiency would not provide information about the fish’s performance but could be employed in situations where the fish undergoes accelerations, such as, for example, evasive maneuvers. The main question is, therefore, what would be an appropriate way to define useful power to quantify the hydrodynamic efficiency of the fish from a mechanical perspective, as well as to understand which parameters influence it.

In this study, the hydrodynamic swimming efficiency of a fish is presented using the definitions of propulsive efficiency $\eta_{P}$ , quasi-propulsive efficiency $\eta_{QP}$ , and the power coefficient $C_{P}$ , respectively (see Equation (5)). The main difference between these definitions lies in the calculation of the useful power $P_{u}$ .

(5) $$\eta_{P}=\frac{\bar{F_{T}}U}{\bar{P_{t}}};\;\eta_{QP}=\frac{RU}{\bar{P_{t}}};\;\bar{C_{P}}=\frac{\bar{P_{t}}}{0.5\rho U^{3}L^{2}}.$$

The propulsive efficiency $\eta_{P}$ calculates the useful power using the propulsive thrust force $\bar{F_{T}}$ , which is the force in the direction of motion (i.e., opposite to the flow). The main drawback of this definition is the calculation of propulsive thrust, both experimentally and numerically, due to the decoupling of the longitudinal force into its thrust and drag components.

On the other hand, the quasi-propulsive efficiency $\eta_{QP}$ proposed by Maertens et al. [8] defines useful power as the power required to tow a fish in a straight line at a given velocity, $\bar{P_{u}}=RU$ . Here, *R* represents the resistance force of the fish as a rigid body without deformation, as well as being towed at the velocity *U* without the consideration of any propulsive components.

Finally, the power coefficient is a dimensionless measure of the swimming power consumption (Equation (5)), where ρ is the fluid density, and *L* is a characteristic length of the problem that typically uses the fish’s length. However, for example, Liu et al. [34] employed the area of the caudal fin to assess the efficiency of a fish with different tail geometries.

After presenting the efficiency measures and fish power consumption, the time evolution longitudinal force $F_{x}\left(t\right)$ was defined in Equation (6). The forces acting on the fish were computed by integrating the pressure and the viscous force on the fish surface *S*, where *p* is the pressure, $\tau_{ij}$ is the components of the deviatoric part of the stress-tensor, and $n_{j}$ is the unitary normal vector components at the fish surface.

(6) $$F_{x}\left(t\right)=\int_{S}\left(-pn_{x}+\tau_{xj}n_{j}\right)dS.$$

As previously mentioned, the hydrodynamic force decomposition (drag and thrust) in undulating fish swimming is a complex problem due to the force being a periodic function with a zero mean. In this work, we employed the method reported by Borazjani and Sotiropoulos [15] to separate the longitudinal forces into positive and negative parts, thereby distinguishing between thrust $F_{T}$ and drag $F_{D}$ , such as in Equation (7). Here, the thrust was computed through the mean of the positive part of the force signal, and the swimming drag was computed as the mean of the negative part (Equations (9) and (11)).

(7) $$F_{x}\left(t\right)=F_{T}\left(t\right)+F_{D}\left(t\right).$$

The thrust forces act against the flow and are defined as positive, while the drag represents the forces in the direction of the flow and is defined as negative. Both forces were decomposed into pressure and viscous components using the subscripts *p* and *v*, respectively (Equations (8) and (10)), as proposed by Borazjani and Sotiropoulos [15]. The temporal dependence is eliminated in the subsequent development to simplify the following equations.

(8) $$F_{T}=F_{T_{p}}+F_{T_{v}},$$

(9) $$F_{T}=\underset{F_{T_{p}}}{\underset{︸}{\frac{1}{2}\left[\int_{A}-pn_{x}dA+\left|\int_{A}pn_{x}dA\right|\right]}}+\underset{F_{T_{v}}}{\underset{︸}{\frac{1}{2}\left[\int_{A}\tau_{xj}n_{j}dA+\left|\int_{A}\tau_{xj}n_{j}dA\right|\right]}},$$

(10) $$F_{D}=F_{D_{p}}+F_{D_{v}},$$

(11) $$F_{D}=\underset{F_{D_{p}}}{\underset{︸}{\frac{1}{2}\left[\int_{A}-pn_{x}dA-\left|\int_{A}pn_{x}dA\right|\right]}}+\underset{F_{D_{v}}}{\underset{︸}{\frac{1}{2}\left[\int_{A}\tau_{xj}n_{j}dA-\left|\int_{A}\tau_{xj}n_{j}dA\right|\right]}}.$$

Consequently, thrust forces can be separately computed from drag forces, thereby allowing the determination of the proposed propulsive efficiency in Equation (5). The longitudinal forces acting on fish in Equation (7) are normalized by employing the resistance force, *R*, which is computed for a non-deformed fish ( St=0 ) and results in the net force coefficient $C_{F}\left(t\right)$ , thrust coefficient T(t) , and swimming drag coefficient D(t) .

(12) $$C_{F}\left(t\right)=\frac{F_{x}\left(t\right)}{R};\;T\left(t\right)=\frac{F_{T}\left(t\right)}{R};\;D\left(t\right)=\frac{F_{D}\left(t\right)}{R}.$$

The dimensionless force coefficients, which are calculated using the drag (resistance) force *R*, establish a relationship between the force generated by the fish’s undulatory motion and the force acting on the fish when it behaves as a rigid body. In addition to the dimensionless coefficients presented in Equation (12), we defined the coefficients $C_{R}$ and $\bar{C_{T}}$ using the traditional way of defining hydrodynamic coefficients, such as

(13) $$C_{R}=\frac{R}{0.5\rho U^{2}L^{2}};\;\bar{C_{T}}=\frac{\bar{F_{T}}}{0.5\rho U^{2}L^{2}}.$$

Finally, note that the drag and thrust coefficients defined in Equation (13) allow us to express the propulsive and quasi-propulsive efficiencies as a function of the traditional hydrodynamic coefficients, $C_{R}$ , $\bar{C_{T}}$ , and $\bar{C_{P}}$ (Equation (14)). From now on, the horizontal bar on the coefficients denotes the temporal average coefficient value over a swimming cycle.

(14) $$\eta_{P}=\frac{\bar{C_{T}}}{\bar{C_{P}}};\;\eta_{QP}=\frac{C_{R}}{\bar{C_{P}}}.$$

### 2.3. Numerical Setup

The open-source software OpenFOAM^®^ was employed along with a dedicated module for reproducing the fish’s swimming motion through a dynamically adaptive mesh discretization and the mesh generation utility snappyHexMesh to create a hexahedral mesh. Simulations were carried via an URANS approach within the framework of the transition turbulence model *k*- ω -SST, which is also called γ - $Re_{\theta }$ -SST [68]. This model adjusts the parameters in regions where the Reynolds numbers are typically below a critical threshold with a higher frequency of occurrence within the boundary layers. This methodology was chosen for its ability to accurately capture the vortex dynamics in the wake flow and to calculate the hydrodynamic forces involved in propulsive movement.

**Figure 3:**
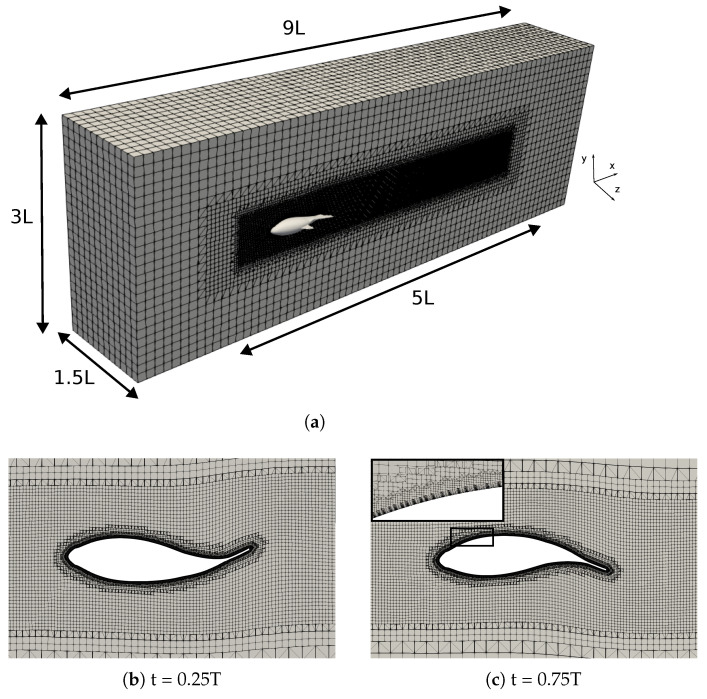
(**a**) Computational domain and numerical mesh (2.2 million elements); (**b**,**c**) adaptive meshes at two different times in a cycle, as well as a detailed view of the prismatic elements in the boundary layer.

The computational domain employed was a prism measuring 3L×3L×9L , where the fish were positioned 2L downstream from the inlet. The numerical mesh consisted of a refinement region to ensure accurate flow resolution around the fish and in the wake, as depicted in Figure 3. Additionally, the prismatic elements were generated to replicate the boundary layer on the fish’s surface, thus maintaining the non-dimensional distance from the wall $y^{+}$ close to unity, as required by the turbulence model.

A study on grid independence was conducted by systematically refining the fish surface and near-wake region. Three structured grids were created with node counts of 1.1, 2.2, and 4.6 million. Table 1 provides a summary of the grid node count, the size element around the fish $\Delta_{x}$ , the $y_{ave}^{+}$ value, the mean thrust force $\bar{F_{T}}$ (Equation (9)), and the relative errors on $\bar{F_{T}}$ between the consecutive grids. The table shows that the relative error between the medium and fine grids was less than 1% in the mean values of the thrust forces, thus indicating a spatially converged solution. Consequently, the medium grid was chosen to run the simulations because it enables practical resolutions that balance accuracy and computational cost.

**Figure 4:**
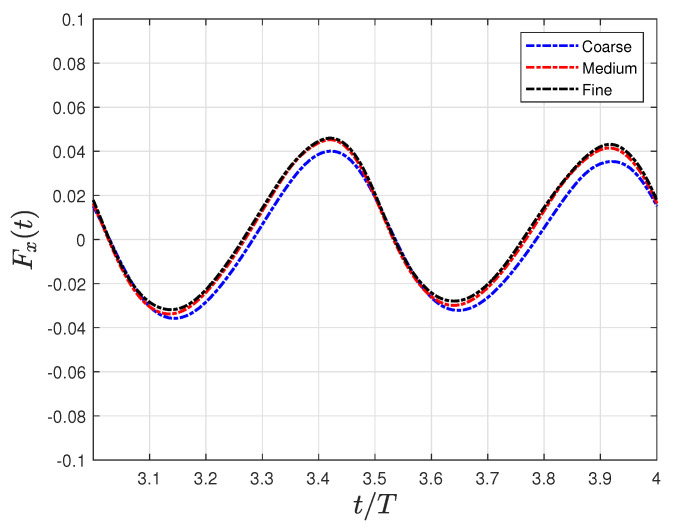
Grid independence study concerning the time evolution force in the flow direction $F_{x}\left(t\right)$ using three distinct grids: coarse grid ( $1.1\times 10^{6}$ ), medium grid ( $2.2\times 10^{6}$ ), and ( $4.6\times 10^{6}$ ) fine grid.

Figure 4 supports the results of Table 1, where the instantaneous net force in the flow direction was compared using three different grids. Finally, a time step of $10^{-4}$ was used in all simulations. This specific time step value was selected after conducting a time-dependent study, which demonstrated that, for time step values less than $10^{-4}$ , errors of less than 0.5% were observed in the calculation of forces.

On the inlet surface, the imposed boundary conditions included a uniform velocity of *U* and a turbulence intensity of 5%. At the outlet surface, the pressure was specified as the reference value. The non-slip condition was applied to the fish’s surface, and the boundary movement was constrained according to its kinematic equations. A free-slip condition was used on the sidewalls. All residuals computed were found below $10^{-5}$ .

Another study involving numerical simulations of lambari using this same methodology was conducted by Macias et al. [13]. The authors developed a set of simulations to compute the fish swimming equilibrium frequency ( $f^{*}$ ) at different Reynolds numbers and, consequently, the equilibrium Strouhal number ( $St^{*}$ ). Note that the equilibrium condition (^∗^) guarantees the longitudinal force balance.

In this research, a systematic variation of the main parameters governing the fish locomotion, Re , St , and Slip , were undertaken to investigate their effects on various efficiency metrics, wake flow patterns, and vortex production. To achieve this goal, the Reynolds number was systematically varied within a range of ( $10^{3}$ – $10^{6}$ ), and, for each specific case, an equilibrium condition was identified. For each Reynolds number, the single equilibrium frequency $f^{*}$ was determined, which, consequently, defined an equilibrium Strouhal number, denoted as $St^{*}$ . This equilibrium condition ensured that the fish was neither self-propelled nor dragged by the surrounding flow, thus achieving a balance between the thrust and drag forces in the direction of the flow. Additionally, for select configurations, the Slip number was also systematically altered to gain insight into its impact on the wavelength. The methodology employed in this study utilized an inertial reference frame, which moved at a constant velocity of *U* and was attached to the fish.

In Appendix A, Table A1 and Table A2 provide a summary of the parameters for the main configuration’s run. These parameters include the Reynolds number, flow velocity, wavelength, frequency, Strouhal number, and slip number. Initial simulations were conducted to determine the equilibrium frequency values by monitoring the balance of longitudinal forces (Table A1), which was followed by a series of simulations for equilibrium configurations that varied in wavelengths in the kinematic equation (Table A2).

## 3. Results and Discussion

### 3.1. Equilibrium of the Strouhal Number and Reynolds Number

Initially, a systematic variation in the swim frequency was conducted for each Reynolds number, with the computation of the equilibrium Strouhal number was conducted in each case. It should be noted that in our simulations the fish were assumed as steadily swimming and the flow velocity was constant. As a result, an equilibrium configuration was achieved when the mean force coefficient in the flow direction was equal to zero, i.e., ${\bar{C}}_{F}=0$ , thereby signifying a balance between the drag and thrust forces. In this work, we employed a sign convention, where a positive mean force coefficient ${\bar{C}}_{F}>0$ indicates net thrust forces (longitudinal forces opposing the flow direction), while a negative value ${\bar{C}}_{F}<0$ signifies net drag forces (longitudinal forces in the same flow direction).

**Figure 5:**
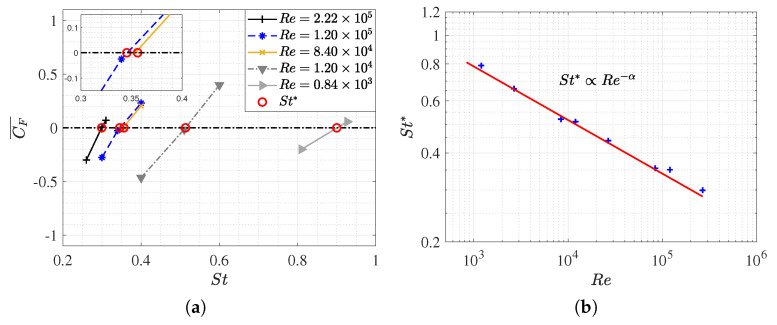
(**a**) Mean force coefficient $\bar{C_{F}}$ as a function of St for different Re ; (**b**) the equilibrium Strouhal number $St^{*}$ dependence on Re , as represented in the power law $St^{*}\propto Re^{\left(-\alpha \right)}$ . The numerical data are represented by the symbol ( + ), and the curve fitting is shown by the red line, where $St^{*}=2.7Re^{\left(-0.18\right)}$ .

Figure 5a illustrates the ${\bar{C}}_{F}$ curves as a function of St for some of the Reynolds numbers under investigation. From these curves, the value of $St^{*}$ is estimated through the linear interpolation of the pair of points on the ${\bar{C}}_{F}\times St$ curve that contains it [13]. After determining the frequency value that approximates the null mean force ${\bar{C}}_{F}∼0$ , the simulation is executed to reach the equilibrium configuration. The data plotted in Figure 5a is summarized in Table A1.

Within the scope of our study, a single critical Strouhal number was identified for each Re , and it was observed that ${\bar{C}}_{F}$ , which monotonically increased as the Strouhal number rose. This was likely due to fish needing to swim at higher frequencies to counteract the enhanced viscous forces associated with lower Reynolds numbers. Figure 5b displays the estimated data of $St^{*}$ for each Reynolds number, thereby showing that fish locomotion dynamics conform to a power–law relationship that is predicted to data fitting, where $St^{*}=2.7Re^{\left(-0.18\right)}$ . This aligns with the Strouhal and Reynolds dependencies of $St\propto Re^{\left(-\alpha \right)}$ proposed by Gazzola et al. [10]. The data plot in Figure 5b is documented in Table A2, where the simulations that were conducted when the fish swam in the equilibrium condition with λ/L =0.95 are summarized. Furthermore, Table A2 presents the additional configurations that involve changes in λ/L , which will be analyzed later.

In both figures, it is apparent that the influence of the Reynolds number Re on the Strouhal number St diminishes at higher Re values. Furthermore, for Reynolds numbers exceeding $10^{5}$ , Strouhal values around 0.3 were observed, which is consistent with the predictions made by the authors in their research. It is remarkable that this is the actual range in which the minnow is found swimming in nature.

### 3.2. Hydrodynamic Force Coefficients: Swimming Drag and Thrust

**Figure 6:**
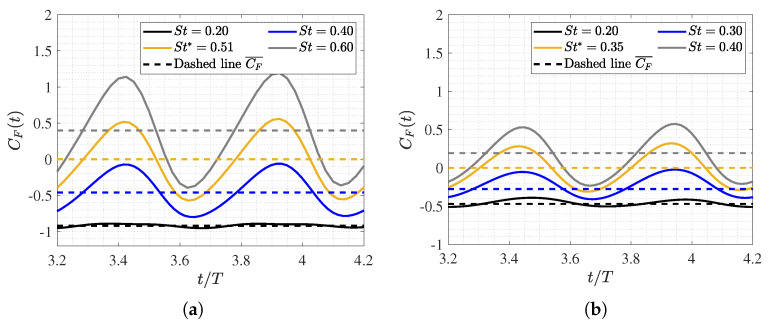
Temporal evolution of the force coefficient during a swimming cycle (i.e., dimensionless time scaling when using the period *T*). The force coefficient $C_{F}\left(t\right)$ quantifies forces in the direction of flow and is normalized by the non-deformed fish’s drag force *R*. (**a**) $Re=1.2\times 10^{4}$ and (**b**) $Re=1.2\times 10^{5}$ .

Figure 6 illustrates the time evolution of the force coefficient, which is denoted as $C_{F}\left(t\right)$ (Equation (12)), in a dimensionless swimming cycle t/T for two different Reynolds numbers, i.e., $1.2\times 10^{4}$ and $1.2\times 10^{5}$ , at various Strouhal numbers, where only the swimming frequency *f* is varied. The data plotted in the figure is summarized in Table A1.

As shown in Figure 5a, the equilibrium configurations ( $St=St^{*}$ ) exhibit a mean longitudinal force coefficient of zero when the fish swims at a constant velocity. In situations outside of equilibrium, the fish may experience acceleration ( $\bar{C_{F}}>0$ ) or deceleration ( $\bar{C_{F}}<0$ ), which are characterized by Strouhal numbers that are higher ( $St>St^{*}$ ) or lower ( $St<St^{*}$ ) than those at equilibrium, respectively. Therefore, as expected, it was noted that, depending on the Strouhal number, the fish will experience net thrust or drag forces accordingly.

Both figures depict the dynamics of fish swimming. These are characterized by two peaks in the force coefficient per cycle, which is a consequence of the tail beat (two tail strokes per swimming cycle). In all cases, the amplitude of the force coefficient signal, as well as its temporal mean, monotonically increased as the Strouhal number rose, thus indicating that higher swimming frequencies are associated with greater thrust. In fact, faster movements of the caudal fin enhance the downstream fluid emission, thus contributing to the fundamental swimming mechanism of carangiform fish in generating thrust for propulsion.

On the other hand, the influence of the Reynolds number is evident in the fact that the excursion amplitude of the force signal is more substantial and extended at lower Reynolds numbers. This is due to the fish having to overcome greater drag forces in a more viscous flow. Consequently, the required tail beat frequency for a fish to experience any form of thrust is significantly higher as the Re decreases, as illustrated in Figure 5a,b.

As previously mentioned, calculating the swimming drag and thrust forces on a body with undulatory motion is a complex problem. In this study, we employed the method proposed by Borazjani and Sotiropoulos [15] to decouple the net longitudinal force into its drag and thrust components by taking the respective mean values of the signal, both positive and negative, for each of these forces.

**Figure 7:**
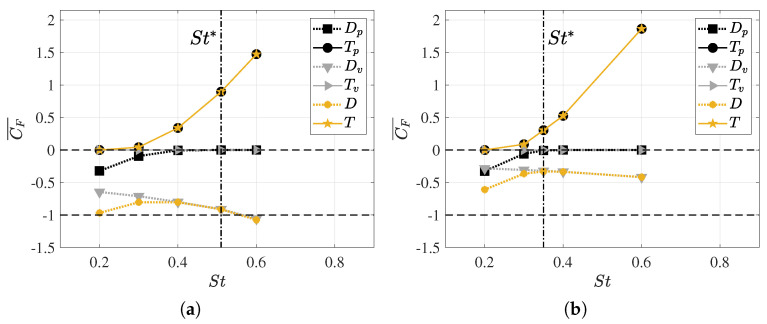
Mean values of the decoupled thrust and drag coefficients as a function of the Strouhal number, where the net thrust (*T*) and drag (*D*) values are displayed along with their pressure components ( $T_{p}$ and $D_{p}$ ) and viscous components ( $T_{v}$ and $D_{v}$ ), respectively. All force values are averaged over one swimming cycle. (**a**) $Re=1.2\times 10^{4}$ and (**b**) $Re=1.2\times 10^{5}$ .

Figure 7 presents the mean values (averaged over one swimming cycle) of the thrust coefficients *T* and drag coefficients *D*, as well as their components due to pressure forces ( $T_{p}$ and $D_{p}$ ) and viscous forces ( $T_{v}$ and $D_{v}$ ), for Reynolds numbers of $1.2\times 10^{4}$ and $1.2\times 10^{5}$ , respectively.

In analyzing the dependence on the Reynolds number, it was observed that the form drag $D_{p}$ did not exhibit a significant difference in behavior for both configurations due to pressure forces. Furthermore, it showed an asymptotic tendency toward zero for Strouhal values between 0.3<St<0.4 . Furthermore, it is noteworthy that, for a Strouhal number of St=0.2 , the fish’s dynamics involve pure drag since there is no thrust force component at this frequency, thus resulting in an induced pressure field that produces only drag forces.

In the case of frictional drag $D_{v}$ , due to viscous forces, a monotonic increase was observed with rising undulatory motion, i.e., with the swimming frequency and, consequently, St . However, this trend was found to be smoother at higher Reynolds numbers since shear stresses on the fish’s surface decreased due to greater inertial forces. To achieve a self-propulsive motion, the lambari generates thrust forces that balance the drag forces, and this originates from the pressure field that results from undulatory motion. As seen in Figure 6b, the thrust forces began to appear at St>0.3 , and the fish’s undulatory motion significantly amplified these forces, thus counteracting the increase in swimming drag. Thus, for high swimming frequencies, net thrust forces were computed.

**Figure 8:**
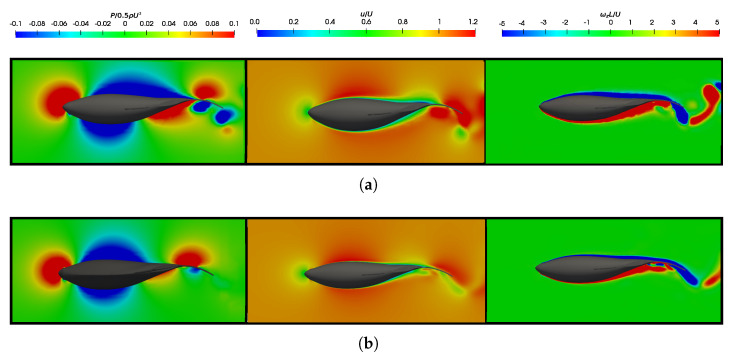
Dimensionless pressure and velocity fields to fish swimming at t/T=4.0 are as follows: (**a**) $St^{*}=0.51$ ; $Re=1.2\times 10^{4}$ ; and (**b**) $St^{*}=0.35;Re=1.2\times 10^{5}$ .

To contribute to the discussion on force production, Figure 8 displays pressure, velocity, and vorticity fields for equilibrium Strouhal configurations at both Reynolds numbers. The boundary layer confined the velocity curl near solid surfaces, which is visualized as high vorticity regions around the fish body. Figure 8a shows the results for the lower Reynolds number, which presents a thicker boundary layer with a higher skin factor. Consequently, this situation corresponds to a regime characterized by higher frictional drag forces [69].

**Figure 9:**
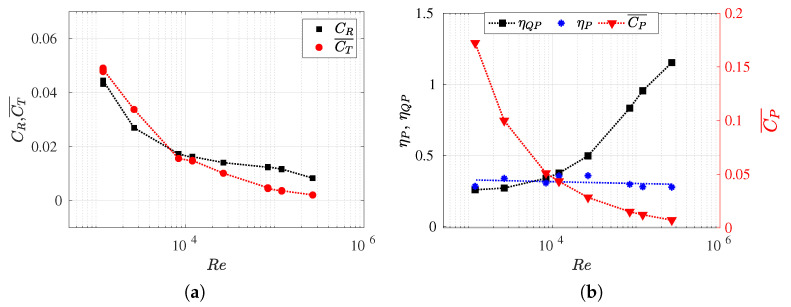
(**a**) Drag resistance coefficient (non-deformed fish) $C_{R}$ and thrust swimming (drag swimming) coefficient $\bar{C_{T}}$ evolution within the Reynolds numbers; (**b**) power consumption coefficient $\bar{C_{P}}$ , propulsive efficiency $\eta_{P}$ , and quasi-propulsive efficiency $\eta_{QP}$ versus Reynolds numbers. All the configurations correspond to equilibrium situations at λ/L=0.95 , which are summarized in Table A2.

In analyzing the pressure field, it was noticeable that its overall topology is similar in both situations. However, it appeared more prominently at lower Reynolds numbers owing to the higher swimming frequencies. A quantitative analysis from Figure 7a,b estimates that, in equilibrium situations, the resulting mean pressure forces in a cycle are $T_{p}=0.9$ and $T_{p}=0.3$ for $1.2\times 10^{4}$ and $1.2\times 10^{5}$ , respectively. This clearly indicates the necessity for the fish to exert greater efforts to achieve the condition of dynamic force balance at lower Reynolds numbers as it must overcome higher frictional forces. In terms of energy, Figure 9b illustrates that the power consumed in one cycle, which is represented by $\bar{C_{P}}$ , was lower for higher Reynolds numbers. Nevertheless, the propulsive efficiency showed similar values. Therefore, in this context, the energy expenditure will be higher at lower Reynolds numbers, but the propulsive efficiency of swimming will not undergo significant changes. All of these aspects will be discussed in more detail in the following section.

### 3.3. Reynolds Number Effect on Efficiency Metrics

In this section, we present the propulsive efficiency ( $\eta_{P}$ ), quasi-propulsive efficiency ( $\eta_{QP}$ ), and the power coefficient ( $\bar{C_{P}}$ ) as functions of the Reynolds number (refer to Figure 9b).

In addition, we also investigated the influence of the Reynolds number on the drag coefficient ( $C_{R}$ ) for non-deformed fish, as well as the mean thrust coefficient ( $\bar{C_{T}}$ ) (see Figure 9a). All the efficiencies and coefficients analyzed were determined for equilibrium configurations and the same wavelength λ/L=0.95 (refer to Table A2). It is important to note that, in situations of force balance, swimming drag values closely approximate thrust coefficient values. Moreover, for this particular body and its kinematics, the form drag is negligible in equilibrium configurations (Figure 6). Thus, the exclusive drag contribution is attributed to skin drag, which results from viscous effects, and it is directly computed through numerical simulations. In these situations, the method employed to calculate the separation of thrust and drag forces introduces no approximation or error.

Firstly, in Figure 9a, it can be observed that the $C_{R}$ exhibits a behavior similar to the drag swimming coefficient for an aerodynamic body, such as an airfoil. It is noted that for lower Reynolds numbers, the drag value for the non-deformed body increases, and this is attributed to the rise in frictional forces in such regimes. On the other hand, for higher Reynolds numbers, the decrease in $C_{R}$ becomes less noticeable. The same phenomenon was observed for the thrust coefficient (or swimming drag coefficient) but in a more pronounced manner. In inertial regimes, the drag progressively decreases approaching a very small value, while in highly viscous flows, the swimming drag $\bar{C_{T}}$ increases considerably. Thus, the fish’s undulatory motion contributes to minimizing drag at high Reynolds numbers and increasing it for lower values, where the swimming frequency is higher in order to balance the forces.

It can also observed that there is a critical Reynolds number value where the curves intersect ( $Re_{cr}∼10^{4}$ ), wherein $C_{R}$ equals $\bar{C_{T}}$ , which means that the drag swimming coefficient achieves the same value as the non-deformed drag coefficient. Consequently, when $Re<10^{4}$ , then $\bar{C_{T}}/C_{R}>1$ . Conversely, when $Re>10^{4}$ , then $\bar{C_{T}}/C_{R}<1$ . Maertens et al. [8] described the ratio $\bar{C_{T}}/C_{R}$ as drag amplification to compare the drag forces that fish experience when stationary and when swimming (i.e., non-deformed and deformed, respectively). Consequently, the drag amplification due to undulating motion can be estimated as the ratio between the propulsive efficiency and the quasi-propulsive efficiency, $\bar{C_{T}}/C_{R}=\eta_{P}/\eta_{QP}$ .

In this work, it was observed that, in equilibrium configurations, for a Reynolds number $Re<10^{4}$ ( $Re<Re_{cr}$ ), the drag swimming is amplified, whereas, at higher Reynolds numbers, the resistance force coefficient exceeds the drag swimming. Therefore, for $Re>Re_{cr}$ , where $\bar{C_{T}}/C_{R}<1$ , the *Bone–Lighthill boundary layer thinning* hypothesis [49] was not corroborated due to the swimming drag being smaller than the rigid body drag.

As observed in Figure 6, for a given Reynolds number, higher undulations lead to an increase in the skin and total drag forces, and this is primarily due to the undulatory motion’s ability to reduce form drag. The effect is significantly more pronounced at lower Reynolds numbers. Additionally, independently, in the equilibrium configurations presented in Figure 9, it was noted that the undulatory motion of a fish, depending on the Reynolds number, can be greater or smaller than the corresponding rigid body drag, as was previously mentioned. It is crucial to note that, in Figure 6a, the skin drag—corresponding to $Re=1.2\times 10^{4}$ —and, consequently, the total drag (since the form drag is 0), takes a value of −1 . This implies that the swimming drag is equal to the rigid body drag as the coefficients *D* and *T* are normalized with the value of the non-deformed drag force. This observation aligns with Figure 9a, where, for Reynolds numbers lower than the critical value, in the equilibrium configuration, $\bar{C_{T}}>C_{R}$ . This phenomenon is related to the increase in viscous forces, which results in a thicker and more developed boundary layer. Consequently, the undulations of the fish’s body further contribute to an increase in viscous drag (skin drag).

In Figure 9b, the results of propulsive efficiencies, quasi-propulsive efficiencies, and power coefficients are presented for each analyzed Reynolds number, which enables comparisons across various flow regimes. Initially, it was observed that propulsive efficiencies $\eta_{P}$ maintain similar values for different Re , $\eta_{P}∼30\%$ . This suggests that, in addition to the challenge of estimating thrust force separately, the information obtained from the definition of propulsive efficiency does not provide relevant insights when the goal is to understand the fish’s performance dependence on the flow regime. It is important to note that, in all the configurations discussed in Figure 9, the fish maintained consistent kinematics where the amplitude function a(x) and the wavelength λ remain constant. Therefore, the only parameter that varied was the equilibrium frequency *f* for each Reynolds number. In the following section, the effects of the wavelength and Slip number will be investigated to observe their impact on the calculation of propulsive efficiency.

The quasi-propulsive efficiency denoted as $\eta_{QP}$ is presented in Figure 9b. It possesses the initial advantage of not requiring the decoupling of longitudinal forces as it considers the drag force of the non-deformed fish in the numerator. Thus, to maximize this efficiency, it suffices to reduce the power consumed in performing the propulsive movement. However, in some instances, the value of $\eta_{QP}$ may exceed unity (i.e., greater than 100%) as it is not strictly an average efficiency. This occurs when situations arise where the power consumed for self-propulsion is less than the power necessary to tow the fish’s rigid body. High values of $\eta_{QP}$ were computed for elevated Reynolds numbers, where $\eta_{QP}>1$ was observed for $Re=2.6\times 10^{6}$ . Therefore, $\eta_{QP}$ was found to be an increasing function of Re , thus highlighting how the carangiform swimming mode was less efficient at low Reynolds numbers and allowed for the evaluation of scale effects (and their dependence on Re ) on swimming efficiency. Furthermore, through the analysis of quasi-propulsive efficiency, the consistency was verified that high $St^{*}$ values are associated with low Re values, thus implying faster lateral undulations and, consequently, higher lateral velocities. Hence, greater lateral power losses and lower efficiency results were obtained.

Finally, the power coefficient appeared to be a somewhat counterintuitive alternative for analyzing swimming efficiency, despite providing a measure of consumed power. In Figure 9b, the values of $\bar{C_{P}}$ are displayed, where they reveal an inverse dependence relationship with Re . The power coefficient exhibited an increasing trend as Re decreased. This was because the power generated by the fish during swimming was found to always be greater as the viscous forces of the flow increased. As mentioned earlier, the fish needed to increase the swimming frequency to overcome the viscous flow.

Similar to Figure 9a, the intersection of propulsive and quasi-propulsive efficiencies at the critical Reynolds number was observed, as shown in Figure 9b. The drag amplification was defined by the ratio between these two efficiency measures. It is at this point where $\bar{C_{T}}=C_{R}$ , and therefore, $\eta_{P}=\eta_{QP}$ . Thus, the value of $\eta_{P}$ , which is independent of Re , is limited to the value of $\eta_{QP}$ when $Re=Re_{cr}$ , i.e., when the fish produces the same swimming drag as rigid body drag.

### 3.4. Slip Number and Wavelength Effect on Efficiency Metrics

In this section, we present results for the coefficients $\bar{C_{T}}$ and $\bar{C_{P}}$ , as well as the propulsive and quasi-propulsive efficiencies. In addition, we explore the influence of the Slip number and wavelength. These findings serve to complement the results discussed earlier.

**Figure 10:**
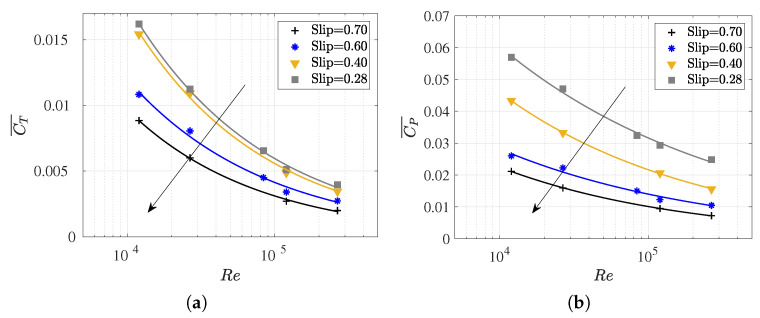
The cycle-averaged (**a**) thrust (or drag swimming) coefficient and (**b**) the power consumption coefficient versus the Reynolds number at various Slip numbers.

**Figure 11:**
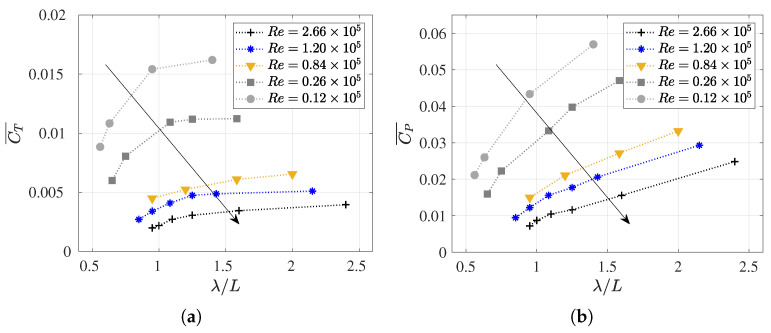
The cycle-averaged (**a**) thrust (or drag swimming) coefficient and (**b**) power consumption coefficient versus dimensionless wavelength ( λ/L ) at various Reynolds numbers.

Figure 10 presents the variations in the coefficients $\bar{C_{T}}$ and $\bar{C_{P}}$ as the Re changes while maintaining the Slip constant. In parallel, Figure 11 elucidates on the behavior of these coefficients for distinct values of the dimensionless wavelength relative to the fish length λ/L , and these were all obtained at the same Reynolds numbers. This deliberate variation allows for a comprehensive exploration of the relationship between the Slip number, wavelength, Reynolds number, and the hydrodynamic coefficients under investigation. The data plotted in Figure 10 and Figure 11 is summarized in Table A2.

As can be observed in Figure 10, the thrust (or swimming drag) and power consumption coefficients increased as the Slip number decreased for a constant Reynolds number. The Slip number represents the ratio between the forward swimming velocity (*U*) and the body undulatory wave phase velocity ( λf ). Therefore, lower Slip numbers define the higher phase velocities of the undulation wave for the same fish velocity *U*, thus implying an increase in the power consumption and the thrust force required for self-propulsion. Additionally, in observing the slopes of the curves, a lesser influence of Reynolds number on the coefficients was noted for higher Slips numbers, primarily in $\bar{C_{P}}$ .

As can be observed in Table A2 for each Reynolds number, a frequency value was set, and the wavelength was systematically varied. Therefore, higher Slip numbers were defined by shorter wavelengths, thus causing the fish to experience more undulations in one cycle. Thus, for a given Reynolds number, higher Slip (smaller λ ) results in lower swimming drag and power consumption coefficients were obtained as the fish underwent more undulations per cycle. This aligned with the findings reported by [14,59,70]. These results are easily observed in Figure 11, where each curve represents a Reynolds number, and the values of $\bar{C_{T}}$ and $\bar{C_{P}}$ increase as the λ/L parameter rises for each Re .

Finally, it is worth noting that for low Re , where viscous effects become increasingly relevant, power consumption and swimming drag exhibit higher values, which further increase with the elongation of the wavelength, thus making the fish stiffer. In this context, it is interesting to observe in Figure 11a, the evolution of $\bar{C_{T}}$ with λ , where an asymptotic trend toward a $\bar{C_{T}}$ for each Re may occur. In any case, this behavior seems irrelevant for the study of carangiform swimmers since Videler and Wardle [67] reported that λ/L falls within the range of 0.98 to 1.1.

**Figure 12:**
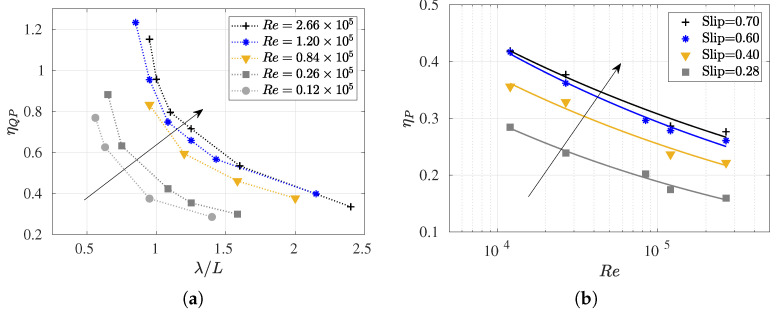
(**a**) Quasi-propulsive efficiency $\eta_{QP}$ versus dimensionless wavelength ( λ/L ) at various Reynolds numbers; (**b**) propulsive efficiency $\eta_{P}$ versus Reynolds numbers at various slip numbers.

From Figure 12a, it is evident that, for a given Reynolds number, $\eta_{QP}$ increases with a decrease in λ/L . This correlation can be attributed to the reduction in the power coefficient for smaller wavelengths, as illustrated in Figure 11. Moreover, it is apparent that, for larger values of λ/L , the variation in $\eta_{QP}$ diminishes, whereas, conversely, for smaller λ/L , minor changes lead to significant fluctuations in $\eta_{QP}$ . This phenomenon arises because, for larger wavelengths where undulations are minimized, the fish become more rigid. Therefore, swimming becomes less efficient as they deviate from the undulatory motion that promotes propulsion.

These findings align with the behavior observed in anguilliform undulating gaits, which are characterized by smaller wavelengths (i.e., higher Slips ), which have been identified as a superior alternative for cruising undulating foils. This is highlighted by studies such as the numerical simulations for 2D foils by Maertens et al. [8] and the investigations supported by Borazjani and Sotiropoulos [33] and Tytell et al. [71], where 3D fish-like eels are studied at Reynolds numbers around $10^{3}$ . In this study, the discussion extends to a broader range of Reynolds numbers $10^{3}$ – $10^{6}$ , which were ascertained by analyzing fish swimming across different flow regimes. This enabled an assessment of the effects of inertial and viscous forces on fish performance, thereby providing a comprehensive understanding of the intricate interplay between the Slip number, efficiency, and the hydrodynamic forces influencing fish locomotion.

**Figure 13:**
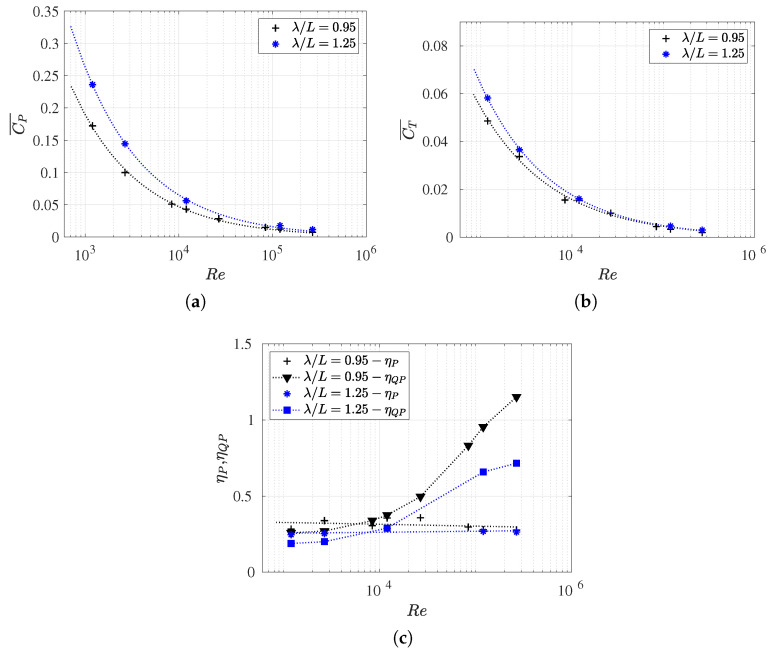
(**a**) The cycle-averaged thrust coefficient; (**b**) the power consumption coefficient; and (**c**) the propulsive and quasi-propulsive efficiencies as a function of the Reynolds number for λ=0.114 and λ=0.150 .

The variation in propulsive efficiency concerning Re and Slip is illustrated in Figure 12b. It is noted that the $\eta_{P}$ increased as the Reynolds number decreased. Upon closer scrutiny of its behavior, it is noteworthy that the efficiency rose with an increasing Slip number, although for higher Slip values, no significant changes were observed in the $\eta_{P}$ values. Generally, the alterations in $\eta_{P}$ with Re were much less noticeable than those for $\eta_{QP}$ , as previously observed in Figure 9b and is further evident in Figure 13c. This figure illustrates the evolution of $\eta_{P}$ and $\eta_{QP}$ against the Reynolds numbers for two different wavelengths ( λ/L=0.95 and λ/L=1.25 ).

It is interesting to note that when the λ is kept constant, $\eta_{P}$ can be considered to oscillate around a constant value for any Reynolds numbers. For λ/L=0.95 , the propulsive efficiency is around $\eta_{P}=0.3$ , and for λ/L=1.25 , it is $\eta_{P}=0.26$ . As expected, both the $\eta_{P}$ and $\eta_{QP}$ values were higher for cases with a shorter wavelength, which is in line with previous results. This is because the fish swimming cruising performance was more efficient in lower wavelengths, which was also akin to the anguiliform undulating gates.

For high values of λ , a change in the trend of the $\eta_{QP}$ curve was observed; if the Reynolds number increases, the $\eta_{QP}$ does not increase at the same rate. This was attributed to the lower values of $\bar{C_{P}}$ and $\bar{C_{T}}$ for high Reynolds numbers, as seen in Figure 13a,b. In both cases, lower λ values exhibited lower values of consumed power and drag swimming, thus resulting in higher efficiencies, as expected.

Finally, it was demonstrated that, for the two wavelengths studied, the efficiency curves, $\eta_{P}$ , and $\eta_{QP}$ intersected for a value of the Reynolds number around $Re_{cr}=10^{4}$ . For the Re , both propulsive and quasi-propulsive efficiencies took on very similar values, with the drag amplification around this value being close to 1 for both analyzed wavelengths. Consequently, this behavior was found to be more dependent on the geometry of the fish than on the kinematics itself. However, it would be appropriate to investigate the variation in the fish wave amplitude in the drag amplification behavior because, from a kinematic point of view, this could be an important variable.

### 3.5. Vortex Dynamics

#### 3.5.1. Leading-Edge Vortex Generation

**Figure 14:**
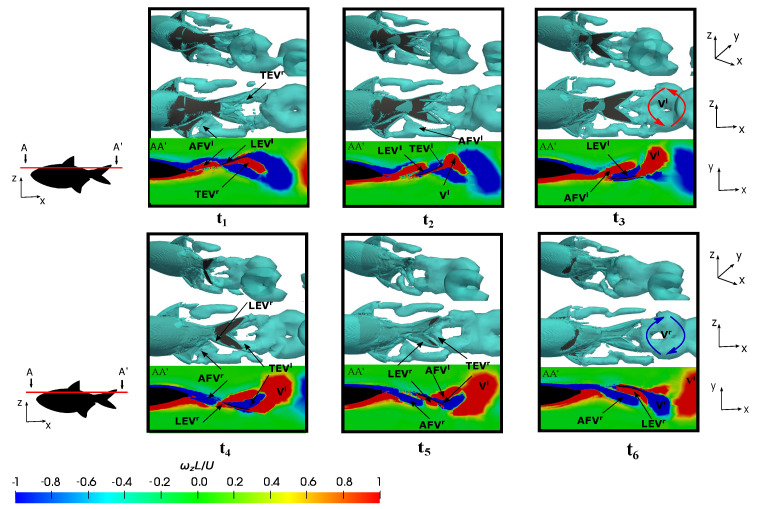
Perspective and lateral views showing the three-dimensional vortex structures employing the variable q-criterion ( q=0.1 ), as well as the frontal view that is presented through the cut-plane AA’ illustrating the dimensionless vortex contour ( $\omega_{z}L/U$ ), to the configuration { $St^{*}=0.35$ ; $Re=1.2\times 10^{5}$ } at six different time instants during a fish swimming period. See the dimensionless time instant $t_{1}$ – $t_{6}$ in Figure 7b, which has the following parameters: $t_{1}=3.30$ , $t_{2}=3.45$ , $t_{3}=3.60$ , $t_{4}=3.75$ , $t_{5}=3.90$ , and $t_{6}=4.05$ .

**Figure 15:**
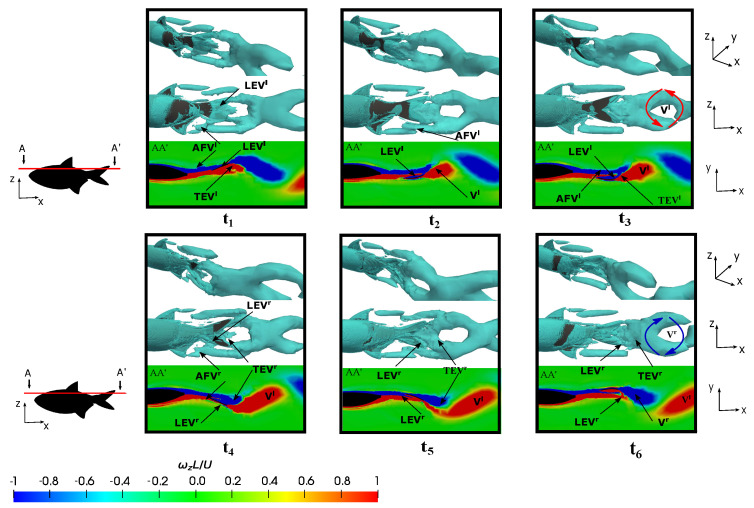
Perspective and lateral views showing the three-dimensional vortex structures employing the variable q-criterion ( q=0.1 ), as well as the frontal view that is presented through the cut-plane AA’ illustrating the dimensionless vortex contour ( $\omega_{z}L/U$ ), to the configuration { St=0.20 ; $Re=1.2\times 10^{5}$ } at six different time instants during a fish swimming period. See the dimensionless time instant $t_{1}$ – $t_{6}$ in Figure 7b, which has the following parameters: $t_{1}=3.30$ , $t_{2}=3.45$ , $t_{3}=3.60$ , $t_{4}=3.75$ , $t_{5}=3.90$ , and $t_{6}=4.05$ .

**Figure 16:**
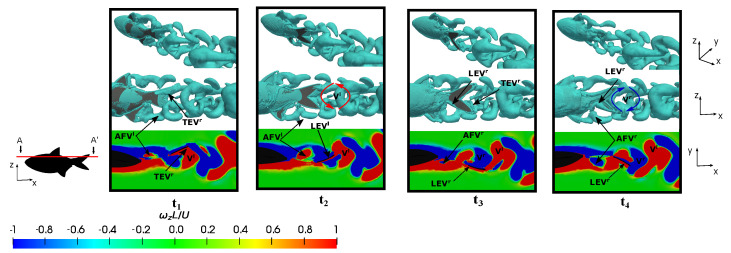
Perspective and lateral views showing the three-dimensional vortex structures employing the variable q-criterion ( q=0.1 ), as well as the frontal view that was presented through the cut-plane AA’ illustrating the dimensionless vortex contour ( $\omega_{z}L/U$ ), to the configuration { $St^{*}=0.51$ ; $Re=1.2\times 10^{4}$ } at four different time instants during a fish swimming period, which had the following parameters: $t_{1}=3.4$ , $t_{2}=3.6$ , $t_{3}=3.8$ , and $t_{4}=4.0$ .

The representation of fish vortex production on the posterior body and caudal fin during a swimming period under various conditions is presented in Figure 14, Figure 15 and Figure 16. Initially, two kinematic scenarios, denoted by $St^{*}=0.35$ and St=0.2 at $Re=1.2\times 10^{5}$ , were examined, as shown in Figure 14 and Figure 15. These were aimed to elucidate on the differences in vortex formation under equilibrium conditions (force balancing) and during pure drag-induced swimming (where the net force is a drag force). Conversely, Figure 16 addresses the equilibrium condition $St^{*}=0.51$ at a lower Reynolds number, $Re=1.2\times 10^{4}$ , where the objective was to investigate the impact of higher viscosity flow on vortex production.

Figure 14 and Figure 15 delineate six time instants ( $t_{1}$ – $t_{6}$ ) within a complete swimming cycle. Plots $t_{1}$ , $t_{2}$ , and $t_{3}$ illustrate a half-stride, leftward tail movement, while figures $t_{4}$ , $t_{5}$ , and $t_{6}$ depict the subsequent rightward tail movement. Following the convention established by Liu et al. [34], the superscripts *l* and *r* were employed to denote the vortices generated during leftward (up-to-down movement) and rightward (down-to-up movement) flapping, respectively.

Figure 14, at $t_{1}$ – $t_{3}$ , elucidates on the development of the leading-edge vortex ( $LEV^{l}$ ) on the right side of the tail, as discerned through the A-A’ cutting plane. In the middle of the flapping motion, precisely at $t_{2}=3.45$ as depicted in Figure 14 at $t_{2}$ , the fish attained a peak force in the force associated with the leading-edge vortex that adhered to the caudal fin surface. This culmination is evident in Figure 7b at $t_{2}$ , where the configuration yielding heightened thrust was pinpointed at the position in $t_{2}=3.45$ . The shedding of the $LEV^{l}$ downstream on the surface was a consequence of the undulatory motion, which subsequently merged with the trailing-edge vortex ( $TEV^{l}$ ) on the caudal fin trailing-edge and the anal fin vortex generated in the preceding half stride ( $AFV^{r}$ ). This amalgamation resulted in the formation of a substantial vortex structure that was emitted into the wake during the subsequent half stroke as a ring vortex ( $V^{r}$ ). It is noteworthy that this negative vortex (depicted in blue) configures itself as a ring vortex by the conclusion of the rightward flapping, while the positive ring vortex ( $V^{l}$ ) is emitted at the conclusion of the leftward flapping. This emission is attributed to the merging of $LEV^{r}$ , $TEV^{r}$ , and $AFV^{l}$ , which are generated in the preceding flappings. Therefore, the fish’s trailing wake induced at the equilibrium frequency manifests as a reverse von-Kármán vortex street, which is characteristic of thrust-force locomotion wherein the positive vortex is situated above the symmetry midplane and the negative one below it. This wake configuration promotes thrust generation due to the high-velocity flow ejected by the fish swimming.

At lower frequencies, e.g., St=0.2 , the formation of the LEV and TEV are evident at the onset of the half stride, along with the presence of the anal and dorsal fin vortices. Subsequently, toward the conclusion of the half stride, the appearance of a ring-vortex shed into the wake becomes apparent. Consequently, the vortex pairs $V^{l}$ and $V^{r}$ originate over the course of a complete cycle. Nevertheless, owing to the reduced lateral velocities, these vortices were emitted more gradually, thus resulting in a lower displacement to the midline. This phenomenon implies that the negative vortices were positioned above the midplane, while the positive counterparts were located below it. The wake conformed to a conventional von-Kármán vortex street, which is characteristic of a purely drag-induced wake, as evident from the force computations presented in Figure 7b at $t_{2}$ .

Observing the evolution of the leading-edge vortex revealed a subtle trend in comparison to the preceding case. As shown in Figure 15 at $t_{6}$ , both $LEV^{r}$ and $TEV^{r}$ were generated. These vortices were subsequently shed during the flapping motion. In this scenario, the size of the $LEV^{r}$ was smaller than that which was generated at $St^{*}=0.35$ , and the enhancement mechanism of the leading-edge vortex was not a pivotal factor in the thrust force generation as the drag forces dominated. Consequently, it was observed that the anal fin vortex did not exert an influence on the caudal fin vortices, thus eliminating the impact of the merged vortex that would otherwise augment the caudal fin strength. Notably, the anal fin vortex was less affected by the shape of the caudal fin during fish swimming, and it shedded without interacting significantly with the caudal fin due to the lower flapping velocity.

Conversely, an investigation was conducted at a lower Reynolds number during fish swimming at the equilibrium frequency in order to elucidate on the influence of the flow regime on vortex production and evolution. Figure 16 illustrates four time instants within a fish swimming cycle, where plots $t_{1}$ and $t_{2}$ present the leftward flapping scenarios, and plots $t_{3}$ and $t_{4}$ showcase rightward flapping scenarios.

Similar to previous cases, the emergence of the LEV and trailing-edge vortex TEV was observed. Notably, $LEV^{r}$ , which was discernible at instants $t_{3}$ and $t_{4}$ , once again manifested in a well-defined tubular form, and it was found to adhere to the caudal fin surface and caused an increase in the thrust-type force production. See how, for lower Reynolds numbers, the vorticity levels in the flow were higher, as well as especially notice how the boundary layer was thicker due to the increase in viscous forces, as mentioned in Figure 8.

**Figure 17:**
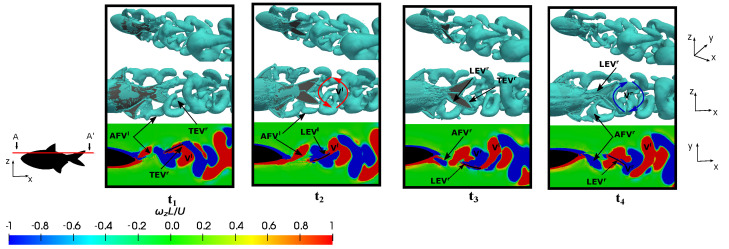
Perspective and lateral views showing the three-dimensional vortex structures employing the variable q-criterion ( q=0.1 ), as well as the frontal view that was presented through the cut-plane AA’ illustrating the dimensionless vortex contour ( $\omega_{z}L/U$ ), to the configuration { St=0.51 ; $Re=1.2\times 10^{5}$ } at four different time instants during a fish swimming period, which had the following parameters: $t_{1}=3.4$ , $t_{2}=3.6$ , $t_{3}=3.8$ , and $t_{4}=4.0$ .

On the other hand, to isolate the influence of the Reynolds number in the analysis and to exclude the effect of the Strouhal number, we presented a case where St=0.51 and $Re=1.2\times 10^{5}$ , as shown in Figure 17. Here, St is held constant and Re is increased. In this scenario, we observed that the dynamics of the vortices induced by the lateral fins and the body were very similar for both Re . It is noteworthy how the morphology of these vortices was affected by the narrow part of the body and caudal fin, and this was also found to a lesser extent for lower Strouhal cases. Additionally, the production of both the LEV and TEV was also observed to be highly similar in both configurations. Furthermore, in both setups, the induced reverse von-Kármán vortex street was notable, and it contributed to the generation of propulsive forces in the fish.

Therefore, based on this analysis, it was observed that the dynamics of the vortices were strongly related to the swimming frequency, where no significant discrepancies were found between the vortices represented in Figure 16 and Figure 17. The main difference between both configurations lay in the higher drag force due to viscous forces in the case of the lower Reynolds number, as can be verified in Figure 7a.

#### 3.5.2. Wake Vortex-Induced Scenarios

The following section delineates the various wake configurations that are induced by a fish swimming, and they are elucidated upon based on parameters such as the Re , St , and Slip numbers.

**Figure 18:**
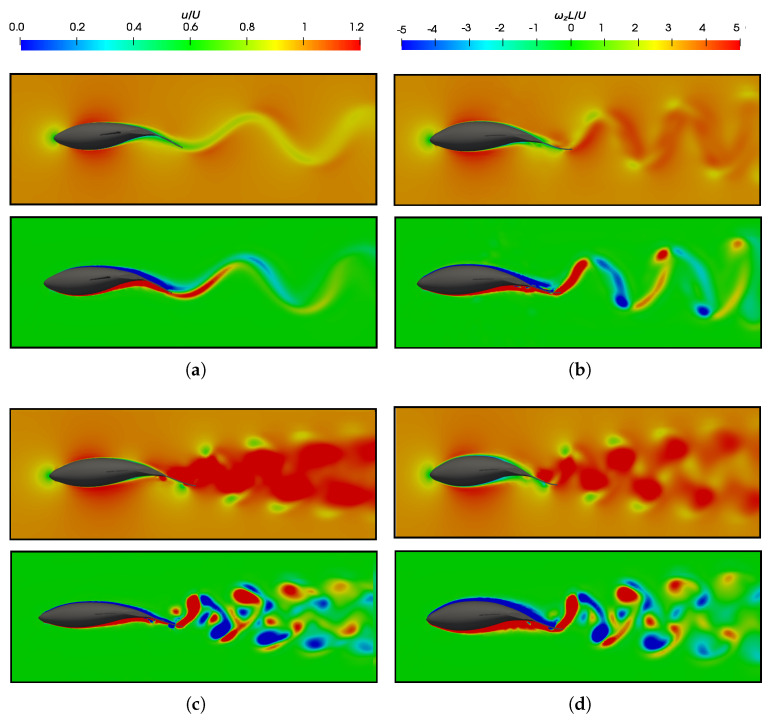
Dimensionless velocity ( u/U ) and vorticity in the *z*-direction ( $\omega_{z}L/U$ ) flow field at λ/L =0.95 with respect to different Strouhal and Reynolds numbers. (**a**) λ/L=0.95 ; St=0.2 ; and $Re=1.2\times 10^{5}$ . (**b**) λ/L=0.95 ; $St^{*}=0.35$ ; and $Re=1.2\times 10^{5}$ . (**c**) λ/L=0.95 ; St=0.60 ; and $Re=1.2\times 10^{5}$ . (**d**) λ/L=0.95 ; $St^{*}=0.51$ ; and $Re=1.2\times 10^{4}$ .

In the main, the wake vortices were compared to the configurations discussed in the previous section, where an examination of the leading-edge vortices was undertaken. Figure 18 illustrates the velocity and vorticity fields in the wake street vortex, which were generated under equilibrium configurations for two distinct Reynolds numbers, namely $1.2\times 10^{4}$ and $1.2\times 10^{5}$ . Furthermore, it also illustrates the wake vortex in a situation defined by pure drag, as described by St=0.2 and $1.2\times 10^{5}$ , as well as complements the discussion of the wake, where another swimming configuration characterized by high Reynolds and Strouhal numbers is presented, as defined by St=0.6 and $1.2\times 10^{5}$ . It is imperative to note that, in all instances, the ratio λ/L is maintained at 0.95.

**Figure 19:**
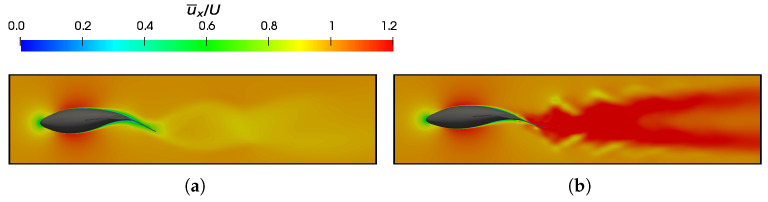
The cycle-averaged dimensionless velocity in the *x*-direction ( ${\bar{u}}_{x}/U$ ) at λ/L = 0.95 and $Re=1.2\times 10^{5}$ . (**a**) St=0.2 and (**b**) St=0.6 .

Fish swimming generates periodic vortices that form a vón Kárman vortex street. In Figure 18a, where $\bar{C_{F}}<0$ (Figure 6b), the wake adopts a traditional vón Kárman vortex street configuration, which exhibits a lower velocity downstream. The vorticity field revealed the emission of negative vortices (which were clockwise and are indicated in blue) above the fish’s midplane, as well as the positive vortices (which were counterclockwise and are represented in red) below it. This outcome provided a more detailed presentation of the vortex street configuration that was previously illustrated in Figure 15. On the contrary, the high Strouhal and equilibrium configurations, as depicted in Figure 18b–d, exhibited a thrust–wake pattern, wherein the velocity within the wake attained higher values. This is in contrast to a drag-type wake, which gives rise to a jet flow behind a fish that propels it forward. For a better understanding, Figure 19 plots the cycle-averaged dimensionless *x*-component of velocity at low and high Strouhal numbers when $Re=1.2\times 10^{5}$ , in which the lowest velocities at St=0.2 and the highest velocities achieved in the wake at higher swimming frequencies at St=0.6 are illustrated.

In terms of vorticity, a reverse vón Kárman street structure was evident, wherein the vortices were arranged with positive vortices above and negative vortices below the midplane, respectively. In addition, at high swimming frequencies (Figure 18c,d), the vortices produced by the body, caudal fin, as well as the pectoral and anal fins, failed to merge, as seen in the cases presented in Figure 18a,b. This is why these high-frequency beating wakes exhibited a more complex topology, where the vortex structures that can be observed did not merge to form a vortex ring.

As a consequence of the variation in the Reynolds numbers in the simulations, the wakes induced by the fish in different investigated scenarios exhibited changes attributable to the increased frictional forces at lower Reynolds numbers. In these cases, an augmentation in the thickness of the fish’s boundary layer was observed, which led to higher drag forces, as depicted in the force coefficients in Figure 7a,b.

In the flows governed by lower Reynolds numbers, fish need to swim at higher frequencies (with a larger St ) to overcome substantial drag forces and to achieve the equilibrium condition, which allows for self-propulsion. Due to the elevated frequency, similar to what happens in the case of high St and Re numbers (Figure 18c), the wake appears more open. This is attributed to the increased phase speed of the undulatory wave ( λf ) and, consequently, the velocity at which the wave propagates. As a result, the vortex rings are emitted more rapidly, thus leading to higher levels of velocity and vorticity in the wake of fish swimming.

**Figure 20:**
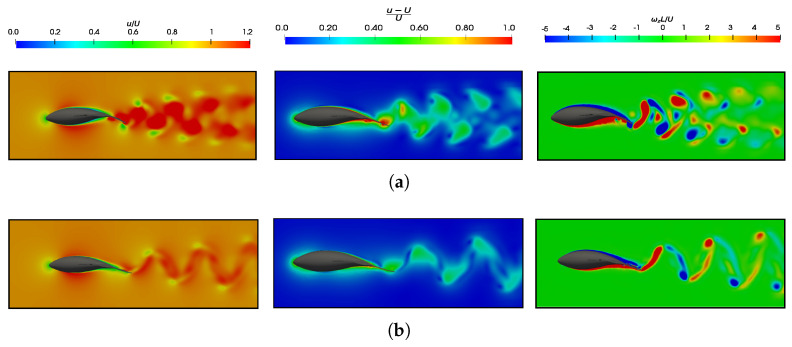
Dimensionless velocities ( u/U ) and ( (u−U)/U ), as well as the vorticity in the *z*-direction ( $\omega_{z}L/U$ ) flow field. (**a**) λ/L=1.25 ; St=0.35 ; and $Re=1.2\times 10^{4}$ . (**b**) λ/L=1.25 ; St=0.51 ; and $Re=1.2\times 10^{5}$ .

**Figure 21:**
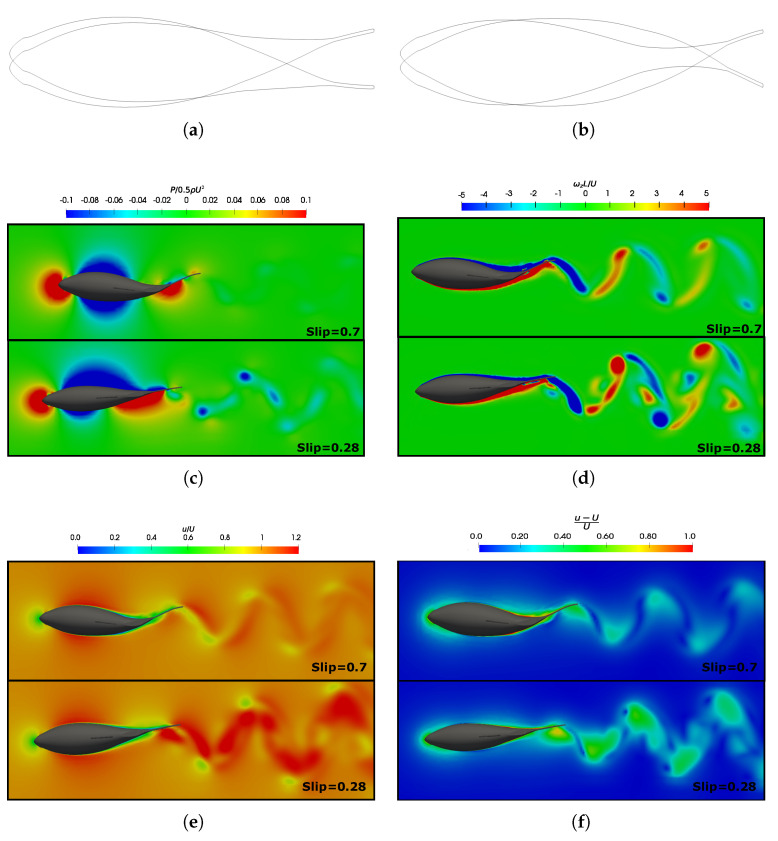
Fish body undulation in two−time instants in a swimming cycle at different slip numbers (and wavelengths). (**a**) Slip=0.28 and λ/L=2.15 ; and (**b**) Slip=0.7 and λ/L=0.85 . Dimensionless flow field at St=0.35 and $Re=1.2\times 10^{5}$ in both slip numbers at the time instance when the caudal fin is at its left-most position: (**c**) pressure ( P/0.5ρU ); (**d**) vorticity in the *z*-direction ( $\omega_{z}L/U$ ); (**e**) velocity ( u/U ); and (**f**) normalized velocity ( (u−U)/U ).

Figure 20 show a comparison of the impact in changing the wavelength on the wake with the situations depicted in Figure 18b,c, and these were found to contribute to a more comprehensive understanding of Figure 13. In the latter, it was observed that, for shorter wavelengths, the values of $\bar{C_{P}}$ and $\bar{C_{T}}$ were lower, thus leading to higher efficiencies. When comparing wakes at the same Reynolds number—as shown in Figure 18d and Figure 20a at $Re=1.2\times 10^{4}$ , as well as Figure 18b and Figure 20b at $Re=1.2\times 10^{5}$ —an increase in velocities in the wake was evident at the highest λ/L ( λ/L=1.25 ). This observation was attributed to the fish displaying a greater phase velocity of body wave undulation, as denoted by λf . At different Reynolds numbers, as shown in Figure 20a, an increase in the phase velocity caused by the higher swimming frequency was demonstrated, which was easily observable from the plot of velocity that was normalized by ((u−U)/U) . Additionally, the fish’s body underwent a less undulatory motion, which behaved more like a rigid body with a movement resembling pitching. Further details on this behavior are elucidated in Figure 21, where the fish’s swimming performance is compared across various Slip numbers.

To compare the effect of the Slip number, we selected the same configuration of Re and St numbers for the highest and lowest Slip values analyzed ( Slip=0.7 and Slip=0.28 ). In Figure 21a,b, the fish geometries are presented at the same instants during a cycle for both of the Slip numbers analyzed. Situations, where the Slip number was higher were characterized by shorter wavelengths, and the fish exhibited a more undulatory motion. In contrast, for lower Slip values, the fish behaved as if they were more rigid, thus emulating movements that were more akin to pitching, as was mentioned earlier.

Therefore, the differences in the deforming bodies made the mechanisms for force production vary. For smaller wavelengths, the formation of vortices at the posterior part of the body and the caudal fin played a crucial role. On the other hand, for cases with higher wavelengths, the pressure difference became the main mechanism for force generation. Thus, Figure 21c illustrates the dimensionless pressure field around the fish, thereby highlighting the notable differences in pressure generation, particularly in the posterior body. When Slip=0.28 , a pressure difference originated on both the pressure and suction sides of the caudal fin, thereby playing a pivotal role in the net thrust force generation. Conversely, when Slip=0.7 , the undulation of the fish’s body resulted in a momentum transfer to the surrounding fluid, which aligned with the observations made by Tekkethil et al. [72].

Through observing the velocity field u/U in Figure 21e, we note that, in the represented situations where velocity, frequency, and viscosity are equal (i.e., identical Reynolds and Strouhal numbers), the velocity in the wake increases when Slip=0.28 due to the higher wave phase undulation speed in that scenario. This behavior is more easily observed in Figure 21f, where the normalized velocity (u−U)/U is plotted. This implication is reflected in Figure 10, where the consumed power is higher. Consequently, both of the efficiencies decreased in this situation, as illustrated in Figure 12b.

**Figure 22:**
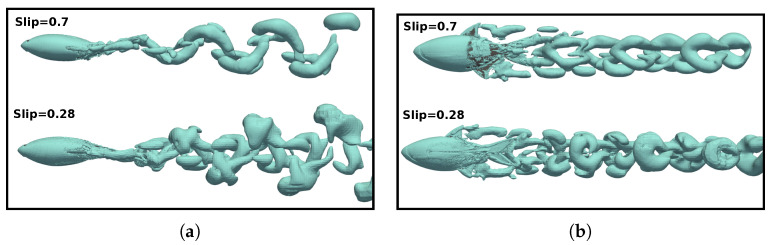
Top (**a**) and frontal (**b**) views showing the three-dimensional vortex structures employing the variable q-criterion ( q=0.1 ) at a time instant t/T=0.25 in a swimming cycle at different Slip numbers (and wavelengths).

From Figure 21d, it is noteworthy that the vorticity magnitude was found to be larger at Slip=0.28 , which resulted in a stronger jet flow behind the fish compared to the Slip=0.7 case. The three-dimensional vortex structures are presented in Figure 22 to enhance the understanding of the wake configuration. In both cases, a double row of ring vortices was induced during fish swimming. However, at lower Slip values, due to reduced body undulation and higher speeds in the wake, the complete fusion of body, tail, and dorsal fin vortices did not occur. Consequently, small fragments of vortices appeared around the vortex rings.

## 4. Conclusions

This study conducted a CFD investigation into the principal dimensionless parameters, Re , St , and Slip , in the hydrodynamics of fish swimming under the typical kinematics of carangiform locomotion. This was achieved by considering a three-dimensional turbulent flow at a constant velocity. The URANS approach with the *k*- ω - SST turbulence closure model was employed for this study, and the fish motion was mimicked by a deformable mesh using kinematic models that were fed by real fish swimming data obtained from the literature. The fish model considered was the steady-swimming lambari.

A power–law relationship was found that correlates with the equilibrium Strouhal values for each Reynolds regime, and this was achieved by noting there was a unique value of $St^{*}$ for each Re , and that lower Re numbers corresponded to a higher $St^{*}$ . As such, the fish required a higher swimming frequency to achieve longitudinal force equilibrium. On the other hand, drag and thrust mean forces were computed out of the equilibrium condition, and it was found that the body undulations significantly increased the skin friction along the body, i.e., the swimming drag, at lower Reynolds numbers. Furthermore, a direct relationship between the mean force coefficient and vortex wake structures was demonstrated; thus, we concluded that thrust forces appear when a fish induces a reverse von Kármán wake due to the downstream high-velocity jet, which enhances fish propulsion. In addition, it is crucial to highlight that the presence of the leading-edge vortex LEV attached to the caudal fin corresponds to the force peak and, consequently, thrust generation. It was also observed that the vortices formed at the posterior part of the fish’s body detached from the body and flow downstream, and they then merged with the caudal fin vortex and produced the vortex rings that form coherent structures in the wake.

In the computation of propulsive efficiency, the literature acknowledges a limitation in calculating thrust. However, in the equilibrium situations examined in this work, the drag component was determined to be 0. Consequently, all the thrust was attributed to pressure components, and all the drag resulted from friction, whereby both forces directly were computed from the simulation. The propulsive efficiency showed no variations with the Reynolds number when the wavelength was held constant. Nevertheless, for lower Slip and longer wavelengths, the propulsive efficiency decreased due to reduced body undulation, thereby resulting in lower swimming drag and consumed power. In contrast, quasi-propulsive efficiency appeared to be a good measure for understanding scale effects as it showed significant variation with the Reynolds number. Higher efficiency values were observed for high Reynolds numbers as the inertia forces decreased, thus leading to lower power consumption by the fish for movement. Additionally, a critical Reynolds number value around $10^{4}$ was identified, where propulsive and quasi-propulsive efficiencies were equal along with drag swimming and resistance force values for the non-deformed fish. In this scenario, for Reynolds numbers lower than the critical value in the equilibrium configuration, we obtained $\bar{C_{T}}>C_{R}$ . This phenomenon was linked to an increase in viscous forces, which resulted in a thicker and more developed boundary layer. Consequently, the undulations of the fish’s body contributed to a further increase in viscous drag (i.e., skin drag).

Finally, it is worth noting that situations with higher Slip values (lower wavelength λ ), a pronounced undulation in the fish body is configured, thereby leading to increased efficiencies and to the approaching of anguilliform gaits that are characterized by smaller wavelengths (higher Slips values). These gaits have been recognized as a superior alternative for cruising undulating foils since they result in lower power consumption due to the lower wave phase velocity of body undulations. In such situations, the wake vortices exhibit lower velocities compared to configurations with smaller Slips numbers, where the fish bodies undergo less undulatory motion resembling a more rigid body with a pitching-like movement. Consequently, for smaller wavelengths, the formation of vortices at the posterior part of the body and the caudal fin play a crucial role, while for cases with higher wavelengths, the pressure difference becomes the primary mechanism for force generation.

This study contributes to the ecological understanding of carangiform swimmers, as well as enhances the knowledge on the influence of the main dimensionless parameters Re , St , and Slip numbers on fish swimming force production and efficiency. Moreover, the conclusions obtained could contribute to the development of bionic engineering. In particular, for an established flow regime, appropriate values for a swimmer’s kinematics, swimming frequency, and wavelength could be imposed to determine the optimal efficiency values. In future work, we will consider evaluating other geometries and swimming patterns for a broader understanding.

## Figures and Tables

**Table 1:** Grid convergence study monitoring the relative error between consecutive grids on the mean thrust force $\bar{F_{T}}$ . Fish swimming configuration: $St^{*}=0.35$ and $Re=1.2\times 10^{5}$ .

Grid	Nodes	$\Delta_{x}$	$y_{\mathrm{ave}}^{+}$	$\bar{F_{T}}$	Error on $\bar{F_{T}}$ (%)
Coarse	$1.1\times 10^{6}$	0.0093*L*	2.48	0.0328	3.8%
Medium	$2.2\times 10^{6}$	0.0051*L*	0.36	0.0341	0.6%
Fine	$4.6\times 10^{6}$	0.0027*L*	0.16	0.0339	-

## Data Availability

Data are contained within the article.

## Appendix A

Details of the systematic variation in the parameters that define the simulations conducted to compute the presented results.

**Table A1:** Simulation parameters where the fish’s swimming frequency has been varied, thereby identifying the equilibrium configuration (*) that ensures longitudinal force balance.

Re	*U* (m/s)	λ /L	*f* ( $s^{-1}$ )	St	Slip
$2.66\times 10^{5}$	2.22	0.95	24.1	0.26	0.81
			27.7 *	0.30 *	0.70 *
			28.7	0.31	0.68
$1.20\times 10^{5}$	1.00	0.95	8.3	0.20	1.052
			12.5	0.30	0.70
			14.6 *	0.35 *	0.60 *
			16.7	0.40	0.53
			25.0	0.60	0.35
$8.40\times 10^{4}$	0.70	0.95	10.4 *	0.36 *	0.60 *
			11.7	0.40	0.53
$1.20\times 10^{4}$	1.00	0.95	8.33	0.20	1.052
			12.5	0.30	0.70
			16.7	0.40	0.53
			21.3 *	0.51 *	0.41 *
			25.0	0.60	0.35
$8.4\times 10^{2}$	0.70	0.95	23.6	0.81	0.26
			26.3 *	0.90 *	0.23 *
			27.1	0.93	0.24

**Table A2:** Simulation parameters where the wavelength (and Slip) for the analyzed Reynolds numbers have been varied.

Re	*U* (m/s)	λ /L	*f* ( $s^{-1}$ )	St	Slip
$2.66\times 10^{5}$	2.22	0.95	27.7	0.30	0.70
		1.00			0.67
		1.10			0.60
		1.25			0.53
		1.60			0.42
		2.40			0.28
$1.20\times 10^{5}$	1.00	0.85	14.6	0.34	0.70
		0.95			0.60
		1.10			0.55
		1.25			0.48
		1.43			0.42
		2.15			0.26
$8.40\times 10^{4}$	0.70	0.95	22.2	0.36	0.60
		1.20			0.48
		1.60			0.36
		2.00			0.28
$2.66\times 10^{4}$	2.22	0.65	40.7	0.44	0.70
		0.75			0.60
		1.10			0.42
		1.25			0.36
		1.60			0.28
$1.20\times 10^{4}$	1.00	0.56	21.3	0.51	0.70
		0.63			0.63
		0.95			0.42
		1.40			0.28
$8.40\times 10^{3}$	0.70	0.95	15.2	0.52	0.41
$2.66\times 10^{3}$	2.22	0.95	61.1	0.66	0.32
		1.25			0.24
$1.20\times 10^{3}$	1.00	0.95	33.4	0.80	0.26
		1.25			0.20
